# Supplementary material for: Association between oral microbiome and seven types of cancers in East Asian population: a two-sample Mendelian randomization analysis
Source: Front Mol Biosci. 2023 Nov 21;10:1327893. doi: 10.3389/fmolb.2023.1327893 (PMC10702768; doi:10.3389/fmolb.2023.1327893)
Supplement: Supplementary file 1 [file DataSheet1.ZIP › Supplementary Materials/MR plots for tongue/tongue═╝/Colorectal cancer/pheno.465_to_colorectal cancer_leave_one_out.pdf]

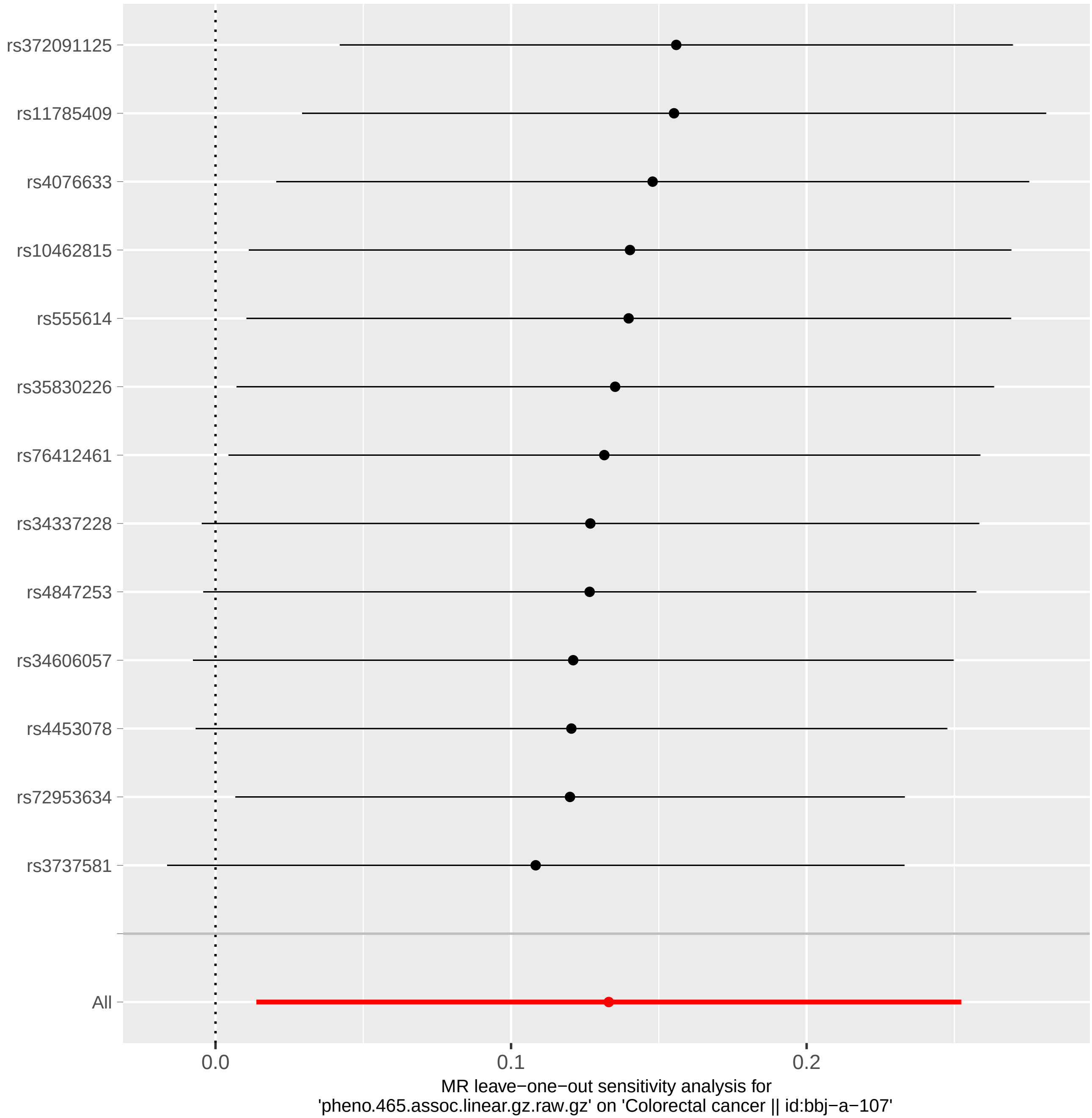

rs372091125
rs11785409
rs4076633
rs10462815
rs555614
rs35830226
rs76412461
rs34337228
rs4847253
rs34606057
rs4453078
rs72953634
rs3737581
All
0.0
0.1
0.2
MR leave−one−out sensitivity analysis for
'pheno.465.assoc.linear.gz.raw.gz' on 'Colorectal cancer || id:bbj−a−107'
